# Hormonal and metabolic effects of polyunsaturated fatty acid (omega-3) on polycystic ovary syndrome induced rats under diet

**Published:** 2014-02

**Authors:** Elaheh Ouladsahebmadarek, Arash Khaki, Sharareh Khanahmadi, Hamidreza Ahmadi Ashtiani, Pooya Paknejad, Mohammad Reza Ayubi

**Affiliations:** 1 Women’s Reproductive Health Research Center, Tabriz University of Medical Sciences, Tabriz, Iran; 2 Department of Pathology, Tabriz Branch, Islamic Azad University, Tabriz, Iran; 3 Department of Biochemistry, Islamic Azad University, Pharmaceutical, Tehran, Iran

**Keywords:** Catalaze (CAT), Follicle stimulating hormone (FSH), Glutathione peroxidase (GPX), Omega-3, Polycystic ovary (PCO), Super oxide dismutase (SOD), Testosterone

## Abstract

***Objective(s):*** PCOS (polycystic ovary syndrome) produces symptoms in approximately 5% to 10% of women of reproductive age (12–45 years old). It is thought to be one of the leading causes of female subfertility. This study aimed to confirm the role of nutrition containing omega-3 (polyunsaturated fatty acid) on control of experimental PCO induced by estradiol-valerat in rats.

***Materials and Methods:*** Wistar female rats (n=40) were allocated into control (n=10) and test groups (n= 30), test group was subdivided into 3 groups: G1, received omega-3 (240 mg/kg/orally/daily); G2 and G3 groups were induced PCO by single injection of estradiol-valerate (16 mg/kg/IM). Group 3 received omega-3 (240 mg/kg/orally/daily) and low carbohydrate feeding for 60 subsequent days; on sixtieth day 5 ml blood samples and ovarian tissues of all rats in the group were removed and prepared for biochemical and hormonal analysis.

***Results:*** Catalase, GPX (Glutathione peroxidase), SOD (Superoxide dismutase) in groups that received omega-3 showed higher levels, but MDA (malondialdehyde) level was significantly decreased (*P*<0.05) in comparison with other experimental groups. Ovarian weights in both experimental and control groups were similar (*P*<0.05). Level of serum FSH (follicle stimulating hormone) was decreased, but level of testosterone was significantly increased (*P*<0.05) in PCO group in comparison with control and omega-3 groups.

***Conclusion:*** Results revealed that administration of omega-3 plus lower carbohydrate food significantly controlled   PCO syndrome and balanced FSH and testosterone.

## Introduction

Polycystic ovary syndrome (PCOS) is a heterogeneous endocrine disorder affecting one in 15 women worldwide. The etiology of this hormonal disorder is unknown, but studies suggest a complex of lifestyle, environmental, and genetic factors. Some metabolic and clinical changes are reported in PCOS cases, including obesity, abnormal menstrual cycle, insulin resistance, cardiovascular diseases, hyperandrogenism symptoms (i.e. hirsutism), acne, and hormonal changes. In this disease, there is elevated level of estrogen but decreased level of progesterone (1-5). Many studies have demonstrated the impact of OS (oxidative stress) on subfertility in both female and male. OS generation in response to hyperglycemia increased in PCOS which may contribute to a pro inflammatory state, so insulin resistance and hyperandrogenism are common in women with PCOS (6, 7).

Since many environmental pollutants cause OS, life style is the most important factor in both sick and healthy individuals (6). OS is significantly associated with excess weight, so weight loss causes improvement of ovulation/menstruation. Low-carbohydrate diets and regular exercise are efficient in weight loss (8, 9). Omega-3 fatty acids (ω−3 fatty acids) are antioxidants found in plant and marine oils. Omega-3 is one of the polyunsaturated fatty acids, the anti-oxidative effect on experimental samples of which, many studies have demonstrated (10, 11). This experiment was designed to evaluate the effect of omega-3 on hormonal and biochemical factor changes in PCO induced rats under diet.

## Materials and Methods

***Animals and diet***

Forty adult 8 week old Wistar albino female rats of 250±10 g were obtained from Animal Facility of Pasture Institute of Iran. Rats were housed in temperature controlled rooms (25°C) with constant humidity (40%–70%) and 12 hr/12 hr light/dark cycle prior to use in experimental protocols. The experimental protocol was approved by the Animal Ethical Committee in accordance with the Guide for the Care and Usage of Laboratory Animals prepared by Tabriz Medical University. All rats were fed a standard diet and water. The daily intake of animal water was monitored at least one week prior to start of treatments in order to determine the amount of water needed per experimental animal. Wistar female rats (n=40) were allocated into control (n=10) and test groups (n=30), test groups subdivided into groups of 3, G1 received Omega-3 (240 mg/kg/orally/daily), G2 and G3 were induced PCO by single injection of estradiol-valerate (16 mg/kg/IM), G3 also received Omega-3 (240 mg/kg) and lower carbohydrate feeding for 60 consecutive days. To demonstrate the effect of carbohydrates on protein utilization, the test diet contained 40% appropriate proteins, 40% carbohydrates, 5% fat, 5% cellulose, and 10 % minerals and vitamins per kg diet. Changes in true protein digestibility, biological value, and net protein utilization were calculated and recorded. For control group, feeding contained 30% appropriate proteins, 50% carbohydrates, 5% fat, 5% cellulose, and 10 % minerals and vitamins per kg diet. On sixtieth day 5 ml blood samples and ovarian tissues of all rats in the groups were removed and prepared for biochemical pathological analysis. Animals were kept in standard conditions; on the 60^th^ day (at the end of the treatment period), blood samples from control and experimental groups were obtained.

***Inducing PCO***

Thirty days before the experimental procedure, twenty rats were each given a single intra muscular (IM) injection of 16 mg/kg estradiol-valerate (Riedeldehaen, Germany) in 0.2 ml oil in order to induce PCO (PCO group). 

***Glutathione peroxidase (GPX) activity measurement in serum***

GPx activity was quantified by following the decrease in absorbance at 365 nm induced by 0.25 mM H_2_O_2_ in the presence of reduced glutathione (10 mM), NADPH, (4 mM), and 1 U enzymatic activity of GR (12).

***Super oxide dismutase (SOD) activity measurement in serum***

The activity of superoxide dismutase (SOD) was measured by following the method of Beyer and Fridovich (13).

***Catalase (CAT) activity measurement in serum***

Serum catalase activity was determined according to the method of Beers and Sizer (14) and measured the decrease in absorbance at 240 nm due to the decomposition of H_2_0 in a UV recording spectrophotometer. The reaction mixture (3 ml) contained 0.1 ml of serum in phosphate buffer (50 mM, pH 7.0) and 2.9 ml of 30 mM H_2_O_2_ in phosphate buffer pH 7.0. Molar extinction coefficient for H_2_O_2_ cm-1 was used for calculation. The specific activity of catalase was expressed as moles of H_2 _reduced per min per mg protein at 240 nm of 40.0 M-1, cm-1 was used for calculation. The specific activity of catalase was expressed as moles of H_2_O_2 _reduced per min per mg protein.

***Malondialdehyde(MDA)concentration*** ***measurement in serum***

 Free radical damage was determined by specifically measuring malondialdehyde (MDA). MDA was formed as an end-product of lipid peroxidation, which was treated with thiobarbituric acid to generate a colored product measured at 532 nm (MDA detection kit from Nanjing Jiancheng Bioengineering Institute, China).

***Determination of blood glucose level***

Blood samples were collected from the tail vein of male rats in all groups. Basal glucose levels were determined by using an automated blood glucose analyzer (Glucometer Elite XL).

***Measurement of FSH hormones***

Serum concentrations of FSH were measured with a two-site chemiluminescence (sandwich) immunoassay using two antibodies specific for the intact FSH molecule. 

***Statistical analysis***

Statistical analysis and test for comparison of data in the control group with the experimental groups was done using the ANOVA. The results were expressed as mean ± SEM (standard error of means). *P*-values less than 0.05 were considered significant and are written in parentheses. 

## Results

***Malondialdehyde (MDA) activity in serum***

Administration of (240 mg/kg/orally/daily) omega-3 for sixty consecutive days significantly decreased malondialdehyde (MDA) concentration in experimental group as compared with the PCOS group (*P*< 0.05), (Figure 1).

***Super oxide dismutase (SOD) activity in Serum***

Administration of (240 mg/kg/orally/daily) omega-3 for sixty consecutive days significantly increased super oxide dismutase (SOD) activity in experimental group as compared with the PCOS group (*P*< 0.05), (Figure 2).

***Glutathione peroxidase (GPX) activity in serum***

Administration of (240 mg/kg/orally/daily) omega-3 for sixty consecutive days significantly increased glutathione peroxidase (GPX) activity in experimental group as compared with the PCOS group (*P*< 0.05), (Figure 3).

***Catalase (CAT) activity in serum***

Administration of (240 mg/kg/orally/daily) omega-3 for sixty consecutive days significantly increased catalase (CAT) activity in experimental group as compared with the PCOS group (*P*< 0.05), (Figure 2).

***Testosterone level in serum***

 Administration of (240 mg/kg/orally/daily) omega-3 for sixty consecutive days significantly decreased testosterone concentration in experimental group as compared with the PCOS group (*P*< 0.05), (Figure 3).

***FSH level in serum***

 Administration of (240 mg/kg/orally/daily) omega-3 for sixty consecutive days significantly increased FSH concentration in experimental group as compared with the PCOS group (*P*< 0.05), (Figure 3).

***Blood glucose level***

Administration of (240 mg/kg/orally/daily) omega-3 for sixty consecutive days significantly decreased blood glucose concentration to 1.75 ± 0.01 g/l in experimental group as compared with the PCOS group (*P*< 0.05), (Figure 4).

**Table 1:** Effect of Omega-3, fatty acids on lipid peroxidation in polycystic ovarian (PCO) rats with lower carbohydrate fed (*P*<0.05

Groups	Control	PCO+ Omega3+Low carbohydrate diet	PCO	Omega-3
MDA (nmol/ml)	4.5±1.2	4.2±1.4	6.2±1.2*	3.0±0.8*

**Table 2:** Effect of Omega-3, fatty acids on antioxidant activities in polycystic ovarian (PCO)rats with lower carbohydrate fed (*P*<0.05)

Groups	Control	PCO+ Omega3+Low carbohydrate diet	PCO	Omega3
SOD (u/ml)	2.58±0.045	2.14±0.05	1.89±0.032*	2.94±0.05
Cat (u/ml)	71.4±16.7	68.3±15.9	56.24±15. 4*	92.3±18.2*
GPX (u/ml)	0.52±0.004	0.48±0.004	0.41±0.002*	0.84±0.005*

**Table 3:** Effect of Omega-3,fatty acids on hormonal levels in polycystic ovarian(PCO)rats with lower carbohydrate fed.(*P*<0.05)

Groups	Control	PCO+ Omega3+Low carbohydrate diet	PCO	Omega-3
Testosterone (ng/ml)	0.45±0.01	1.12±0.05	2.88±0.08*	0.40±0.01*
FSH (ng/ml)	1.15±0.01	0.85±0.01	0.55±0.01*	1.11±0.01

**Table 4:** Effect of Omega-3,fatty acids on blood glucose in polycystic ovarian(PCO)rats with lower carbohydrate fed (*P*<0.05)

Groups	Control	PCO+ Omega3+Low carbohydrate diet	PCO	Omega-3
Blood glucose (nmol/ml)	1.11±0.01	1.75±0.01	2.75±0.01*	1.05±0.01

## Discussion

The impact of dietary composition has been recently evaluated on many diseases such as PCOS; studies showed PCOS outcomes can be affected by diet. An appropriate diet may restore ovulatory function and fertility in obese women with PCOS (15–16). Since dietary carbohydrates increase liver lipogenesis (fat-making) and the activities of enzymes related to lipogenesis, a high-carbohydrate diet induces lipogenesis in adipose tissue (fat tissue) and liver. Hence, in obese women with PCOS, a low-carbohydrate diet could be efficient; besides, recently relation between low carbohydrate diet and modulation of PCOS has been evaluated psychologically, and has been shown to be significant (17-19). 

This study was designed in order to evaluate therapeutic effects of omega-3 along with low-carbohydrate diet on PCO-induced rats. We subdivided rats into a control group and three experimental groups; group 1 just received omega-3 for 60 days; groups 2 and 3 were induced PCOS by estradiol-valerate , group 3 received omega-3 along with low-carbohydrate diet for 60 days as well. After 60 days, biochemical results in group 3 in comparison to the other groups demonstrated positive effects of omega-3 and low-carbohydrate on improvement and normalization of antioxidant enzymes and reduction of oxidative species as well as testosterone. Although ROS have physiological roles in female reproduction, elevated level of ROS by active metabolism and steroidogenesis may cause DNA damage (20–21). Antioxidant enzymes restrict oxidative species to appropriate amounts for physiological processes of the body; antioxidant enzymatic defenses include SOD, GPx, and CAT. Non-enzymatic antioxidants can be represented by ascorbic acid (vitamin C), a-tocopherol (vitamin E), glutathione (GSH), carotenoids, ﬂavonoids, and other antioxidants (22). Superoxide dismutase (SOD), GPx, and catalase have main roles to protect reproductive health, for instance SOD is closely associated with oocyte maturation (23). Oxidative stress and depletion of the antioxidants cause apoptosis (a type of physiological or active cell death) in many systems. Previous works showed that antioxidants prevented apoptosis effectively (24). 

Polyunsaturated fatty acids omega-3(PUFA), especially α-linolenic acid (ALA), eicosapentaenoic acid (EPA), and docosahexaenoic acid (DHA), are bioactive lipids that positively impact signaling pathways involved in the improvement of cardiovascular diseases. Omega-3 may increase high density lipoprotein cholesterol (HDL), but it may decrease the triglyceride levels (25) .In women with PCOS, it can modify the adiponectin (a soluble matrix protein) level, insulin resistance, and lipid profiles, so it has a reducing effect on liver fat content (26). Studies showed modulating effect of PUFAS on hormonal, lipid profiles, and metabolic function in women with PCOS. Omega-3 also has a modulating effect on major risk factors of metabolic syndrome, especially adiposity, dyslipidaemia, insulin resistance, diabetes, hypertension, oxidative stress, and inflammation (27–28). 

This syndrome (PCO) is also caused by hormonal imbalances that have a role in ovulation. PCOS usually causes a decrease in the level of follicle stimulating hormone (FSH) but increases the level of luteinizing hormone (LH). FSH is the hormone that is responsible for stimulating the growth of follicles with maturing eggs in ovaries. After lack of FSH for a long time, the follicles will not mature and release their eggs which results in infertility. Thereafter, the immature follicles in ovaries develop into small cysts and PCO syndrome will happen. Additionally high levels of LH cause the body to produce too much estrogen, androgens (male hormones) , testosterone, and DHEAS (dihydroepiandrosterone sulfate); this imbalance can cause pathological appearances in endometrial tissue and much thickening of the uterus, which can lead to heavy and/or irregular periods (29). Our results indicate that after treatment with omega-3 the mean of FSH was increased, but testosterone was significantly decreased (*P*<0.05).

## Conclusion

he PCO-induced rats receiving omega-3 plus lower carbohydrate diet showed increased antioxidant levels and FSH, but decreased levels of testosterone and MDA, whereas PCO-induced rats not fed omega-3 showed decreased FSH and antioxidant levels but increased levels of testosterone and MDA. Therefore, results of this and the other studies demonstrate omega-3 plus lower carbohydrate feeding could be an efficient therapeutic choice so as to modulate PCOS side effects.

## References

[1] Norman RJ, Dewailly D, Legro RS, Hickey TE (2007). Polycystic ovary syndrome. Lancet.

[2] Teede H, Deeks A, Moran L (2007). Polycystic ovary syndrome: a complex conditions with psychological, reproductive and metabolic manifestations that impact on health across the lifespan. J Free Radic Bio Med.

[3] Lewandowski KC, Cajdler-Łuba A, Salata I, Bieńkiewicz M, Lewiński A (2011). The utility of the gonadotrophin releasing hormone (GnRH) test in the diagnosis of polycystic ovary syndrome (PCOS). J Endokrynol Pol.

[4] Mannerås L, Cajander S, Holmäng A, Seleskovic Z, Lystig T, Lönn M (2007). A new rat model exhibiting both ovarian and metabolic characteristics of polycystic ovary syndrome. J Endocrinology.

[5] González F, Rote N S, Minium J, Kirwan J P (2006). Reactive oxygen species-induced oxidative stress in the development of insulin resistance and hyperandrogenism in polycystic ovary syndrome. J Clin Endocr Metab.

[6] Agarwal A, Aponte-Mellado A, Premkumar BJ, Shaman A, Gupta S (2012). The effects of oxidative stress on female reproduction: a review. Reprod Biol Endocrinol.

[7] Gharagozloo P, Aitken RJ (2011). The role of sperm oxidative stress in male infertility and the significance of oral antioxidant therapy. Hum Reprod.

[8] Sambuca T, Viral H, Haram M (2001). Oxidative stress in polycystic ovary syndrome and its contribution to the risk of cardiovascular disease. Clan Biochem.

[9] Leeman L, Acharya U (2009). The use of metformin inthe management of polycystic ovary syndromeand associated anovulatory infertility: thecurrent evidence. Obstet Gynaecol.

[10] McDonald C, Bauer J, Capra S, Waterhouse M (2013). Muscle function and omega-3 fatty acids in the prediction of lean body mass after breast cancer treatment. Springerplus.

[11] Poudyal H, Panchal SK, Diwan V, Brown L (2011). Omega-3 fatty acids and metabolic syndrome: effects and emerging mechanisms of action. Prog Lipd Res.

[12] Fathiazad F, Khaki A, Nouri M, Khaki AA (2013). Effect of Cinnamon Zeylanicum on serum Testosterone and anti-oxidants levels in Rats. IJWHRS.

[13] Beyer WF Jr, Fridovich I (1987). Assaying for superoxide dismutase activity: some large consequence of minor changes in conditions. Anal Biochem.

[14] Usoh FI, Akpan EJ, Etim EO, Farombi EO (2005). Antioxidant actions of dried flower of Hibiscus sabidariffa L. on sodium arsenite-induced oxidative stress. Pak J Nutr.

[15] Sultana A, Nadir S (2008). Pituitary gonadotropic hormones in women with oligo/amenorrhoea. J Ayub Med Coll Abbottabad.

[16] Moran LJ, Pasquali R, Teede HJ, Hoeger KM, Norman RJ (2009). Treatment of obesity in polycystic ovary syndrome: a position statement of the Androgen excess and polycystic ovary syndrome society. Fertil Steril.

[17] Yancy WS, Olsen MK, Guyton JR, Bakst RP, Westman EC (2004). A lowcarbohydrateketogenic diet versus a low-fat diet to treat obesity and hyperlipidemia. Ann Intern Med.

[18] Rizk AY, Bedaiwy MA, Al-Inany HG (2005). N-acetylcysteine is a novel adjuvant to clomiphene citrate in clomiphene citrate-resistant patients with polycystic ovary syndrome. Fertil Steril.

[19] Galletly C, Moran L, Noakes M, Clifton P, Tomlinson L, Norman R (2007). Psychological benefits of a high-protein, low-carbohydrate diet in obese women with polycystic ovary syndrome—a pilot study. Appetite.

[20] Agarwal A, Gupta S, Sharma RK (2005). Role of oxidative stress in female reproduction. Reprod Biol Endocrinol.

[21] Fujii J, Iuchi Y, Okada F (2005). Fundamental roles of reactive oxygen species and protective mechanisms in the female reproductive system. Reprod Biol Endocrinol.

[22] Valko M, Leibfritz D, Moncol J, Cronin MT, Mazur M, Telser J (2007). Free radicals and antioxidants in normal physiological functions and human disease. Endocrinol Invest Int J Biochem Cell Biol.

[23] Matos L, Stevenson D, Gomes F, Silva-Carvalho JL, Almeida H (2009). Superoxide dismutase expression in human cumulus oophorus cells. J Mol Hum Reprod.

[24] Tsai-Turton M, Luderer U (2006). Opposing effects of glutathione depletion and follicle-stimulating hormone on reactive oxygen species and apoptosis in cultured preovulatory rat follicles. J Endocrinology.

[25] Duda MK (2011). Polyunsaturated fatty acids omega-3 as modulators of intracellular signaling pathways. Postepy Biochem.

[26] Wang SP, Chen YH, Li H (2012). Association between the levels of polyunsaturated fatty acids and blood lipids in healthy individuals. J Exp Ther Med.

[27] Phelan N, O'Connor A, Tun TK, Correia N, Boran G, Roche HM (2011). Hormonal and metabolic effects of polyunsaturated fatty acids in young women with polycystic ovary syndrome: results from a cross-sectional analysis and a randomized, placebo-controlled, crossover trial. Am J Clin Nutr.

[28] Kasim-Karakas SE, Almario RU, Gregory L, Wong R, Todd H, Lasley BL (2004). Metabolic and endocrine effects of a polyunsaturated fatty acid-rich diet in polycystic ovary syndrome. J Clin Endocr Metab.

[29] Tena G, Moran C, Romero R, Moran S (2011). Ovarian morphology and endocrine function in polycystic ovary syndrome. Arch Gynecol Obstet.

